# Three new species of *Picipes* (Polyporales, Basidiomycota) from China

**DOI:** 10.3897/mycokeys.130.183848

**Published:** 2026-03-24

**Authors:** Kai-Yue Luo, Yu-Jin Cui, Si-Yi Feng, Xin Zhang, Yu-Cheng Dai, Guang-Yu Zeng

**Affiliations:** 1 State Key Laboratory of Efficient Production of Forest Resources, School of Ecology and Nature Conservation, Beijing Forestry University, Beijing 100083, China School of Ecology and Nature Conservation, Beijing Forestry University Beijing China; 2 Guangxi Forestry Research Institute, Nanning 530002, China Guangxi Forestry Research Institute Nanning China

**Keywords:** Molecular phylogeny, polyporoid, taxonomy, wood-inhabiting fungi

## Abstract

Three new wood-inhabiting fungi viz. *Picipes abieticola*, *P. brevisporus* and *P. sinodictyopus***spp. nov**. are proposed, based on a combination of morphological features and molecular evidence. *Picipes abieticola* is characterised by eccentrically stipitate basidiomata with fan-shaped pilei, shining pore surface, pores 5–7 per mm and the presence of irregular crystals amongst hyphae, basidiospores measuring 7.5–9.3 × 2.8–3.5 µm and growth on *Abies fabri*. The species *Picipes brevisporus* is characterised by small basidiomata having smaller pores (7–9 per mm), the presence of subulate cystidioles, short oblong ellipsoid basidiospores (3.8–5.7 × 2.3–3.1 µm) and growth on *Miscanthus*. The taxa *Picipes sinodictyopus* are characterised by laterally stipitate basidiomata having auricular to fan-shaped pilei with olivaceous buff pore surface, angular pores and cylindrical basidiospores measuring 5.8–7.3 × 2.3–2.8 µm. The phylogenetic tree inferred from the ITS + nLSU dataset highlighted the placement of the three new species in the genus *Picipes* (Polyporaceae, Polyporales) using Maximum Likelihood and Bayesian Inference methods. Phylogenetically related and morphologically similar species to these three new species are discussed.

## Introduction

Macrofungi are important natural resources, the diversity of the Chinese macrofungi has been extensively studied and a large number of species new to science and many taxa new to China have been recently discovered (Wang et al. 2021, 2023, 2024; Zhang et al. 2023; Zhou et al. 2023, 2026; Dong et al. 2024, 2025; Hongsanan et al. 2025; Song et al. 2025; Wijesinghe et al. 2025; Yang et al. 2025; Zhao et al. 2025).

Polypores, as a major group of macrofungi, play important ecological roles in forest ecosystems (Hibbett et al. 2014), in which 1337 species have been found in the Northern Hemisphere (Justo et al. 2017; Wu et al. 2022a) and 1902 species in the tropical forests of Africa, America and Asia (Zhao et al. 2024). In addition, a total of 4,026 polypores has been found worldwide, suggesting its extremely high species diversity (Zhao et al. 2026).

Recently, on the basis of studying the lentinoid and polyporoid fungi, Zmitrovich and Kovalenko (2016) described a new genus *Picipes* Zmitr. & Kovalenko typified by *Pi. badius* (Pers.) Zmitr. & Kovalenko (= *Polyporus badius*) according to analyses of nLSU, ITS and EF1-α sequences. This genus is characterised by the following features: basidiomata annual, stipitate, hymenophore polyporoid; pilei infundibuliform, covered with a cuticle, without scales, smoke-grey to castaneous or deeply brown; stipe covered with brownish to black cuticle; pores small (more than 5 per mm); hyphal system dimitic with skeletal-binding hyphae; clamp connections present in most species; basidiospores cylindrical to oblong ellipsoid, smooth, hyaline, thin-walled, inamyloid, acyanophilous; on both angiosperm and gymnosperm wood, causing a white rot. *Polyporus badius*, *P. melanopus* (Pers.) Fr. and *P. tubaeformis* (P. Karst.) Ryvarden & Gilb. were segregated from *Polyporus* and transferred into *Picipes* as *Pi. badius*, *Pi. melanopus* (Pers.) Zmitr. & Kovalenko and *Pi. tubaeformis* (P. Karst.) Zmitr. & Kovalenko (Zmitrovich and Kovalenko 2016).

Based on multi-gene phylogenetic analyses, species in *Polyporus* group were proved to be distributed in two different clades viz. picipes clade and squamosus clade (Zhou et al. 2016). The picipes clade has been described as *Picipes* Zmitr. et Kovalenko (Zmitrovich and Kovalenko 2016) and sixteen species with hard basidiomata in dried condition, brownish to black stipes and strongly branched skeleto-binding hyphae were included in this genus (Zhou et al. 2016). Based on the MycoBank database (http://www.mycobank.org, accessed on 6 March 2026) and the Index Fungorum (http://www.indexfungorum.org, accessed on 6 March 2026), 32 species have been accepted in the genus *Picipes* (Zhou et al. 2016; Zmitrovich and Kovalenko 2016; Cui et al. 2019; Bhunjun et al. 2022; Ji et al. 2022; Liu et al. 2024).

In the current study, the species diversity of *Polyporussensu lato* in China was investigated, phylogenetic analyses, based on ITS + nLSU were carried out and three new species matching the concept of *Picipes* are described and illustrated.

## Materials and methods

### Morphological studies

The studied six specimens were collected from nature forests, including Guangxi Autonomous Region, Xizang Autonomous Region and Yunnan Province of China. These are deposited in the Fungarium of the Institute of Microbiology, Beijing Forestry University (BJFC, China). Morphological descriptions are based on field notes and voucher specimens. The microscopic analysis follows Spirin et al. (2017) and Wu et al. (2022b). Freehand sections were made from dried basidiomata and mounted in 2% (w/v) potassium hydroxide (KOH) to observe colour changes. Sections were studied at a magnification of up to 1000× using a Nikon Eclipse 80i microscope and phase contrast illumination. Microscopic features and measurements were made from slide preparations stained with Cotton Blue and Melzer’s reagent. To represent the variation in the size of spores, 5% of measurements were excluded from each end of the range and are given in parentheses. In the description: **KOH** = 5% potassium hydroxide, **IKI** = Melzer’s reagent, **IKI–** = neither amyloid nor dextrinoid, **CB** = Cotton Blue, **CB+** = cyanophilous in Cotton Blue, **CB–** = acyanophilous in Cotton Blue, **L** = arithmetic average of spore length, **W** = arithmetic average of spore width, **Q** = L/W ratios and **n** = number of basidiospores measured from a given number of specimens. Colour terms follow Anonymous (1969) and Petersen (1996).

### DNA extraction, amplification and sequencing

A CTAB rapid plant genome extraction kit-DN14 (Aidlab Biotechnologies Co., Ltd, Beijing) was used to obtain DNA from dried specimens and to perform the polymerase chain reaction (PCR) according to the manufacturer’s instructions with some modifications (Shen et al. 2019; Sun et al. 2020). The internal transcribed spacer (ITS) and large subunit nuclear ribosomal RNA gene (nLSU) were amplified using the primer pairs ITS5/ITS4 and LR0R/LR7 (White et al. 1990; Hopple and Vilgalys 1999, https://sites.duke.edu/vilgalyslab/rdna_primers_for_fungi/).

The PCR procedure for ITS was as follows: initial denaturation at 95 °C for 3 min, followed by 34 cycles at 94 °C for 40 s, annealing at 54 °C for 45 s and extension at 72 °C for 1 min, with a final extension of 72 °C for 10 min. The PCR procedure for nLSU was as follows: initial denaturation at 94 °C for 1 min, followed by 34 cycles of denaturation at 94 °C for 30 s, annealing at 50 °C for 1 min and extension at 72 °C for 1.5 min, with a final extension at 72 °C for 10 min. The PCR products were purified and sequenced at the Beijing Genomics Institute (BGI, China), with the same primers. The newly-generated sequences were deposited in GenBank (Sayers et al. 2024) and listed in Table 1.

**Table 1.** Names, specimen numbers, references and corresponding GenBank accession numbers of the taxa used in the phylogenetic analysis of this study. [New species are shown in bold, * type material].

Species	Sample No.	GenBank Accession No.		References
		ITS	nLSU	
*Datronia mollis*	RLG 6304	JN165002	JN164791	Liu et al. (2024)
*D. stereoides*	Holonen	KC415179	KC415196	Liu et al. (2024)
*Datroniella scutellata*	RLG 9584 T	JN165004	JN164792	Liu et al. (2024)
*Da. tropica*	Dai 13147	KC415181	KC415189	Liu et al. (2024)
*Echinochaete brachypora*	TFMF 24996	AB462321	AB462309	Liu et al. (2024)
*E. ruficeps*	TFMF 15716	AB462310	AB368065	Liu et al. (2024)
*E. russiceps*	TFMF 24250	AB462313	AB462301	Liu et al. (2024)
*Favolus acervatus*	Cui 11053	KU189774	KU189805	Ji et al. (2022)
*F. acervatus*	Dai 10749b	KX548953	KX548979	Ji et al. (2022)
*F. subtropicus*	Cui 4292	NR158408	KX548992	Zhou et al. (2016)
*F. subtropicus*	Li 1938	KX548971	KX548993	Zhou et al. (2016)
*Hexagonia glabra*	Dai 10691	JX569733	JX569750	Ji et al. (2022)
*H. tenuis*	Cui 8468	JX559277	JX559302	Ji et al. (2022)
*Lentinus longiporus*	DAOM229479	AB478879	LC052218	Ji et al. (2022)
*L. longiporus*	WD2579	AB478879	LC052218	Ji et al. (2022)
*L. substrictus*	Wei 1582	KU189767	KU189798	Ji et al. (2022)
*L. substrictus*	Wei 1600	KC572022	KC572059	Ji et al. (2022)
*Microporus affinis*	Cui 7714	JX569739	JX569746	Ji et al. (2022)
*M. flabelliformis*	Dai 11574	JX569740	JX569747	Ji et al. (2022)
*Mycobonia flava*	CulTENN 10256	AY513570	AJ487934	Liu et al. (2024)
*My. flava*	TENN 59088	AY513571	AJ487933	Liu et al. (2024)
*Neodatronia gaoligongensis*	Cui 8055	JX559269	JX559286	Liu et al. (2024)
*N. sinensis*	Dai 11921	JX559272	JX559283	Liu et al. (2024)
*Neofavolus cremeoalbidus*	Cui 12412	KX899982	KX900109	Ji et al. (2022)
*Ne. cremeoalbidus*	TUMH50009	AB735980	AB735957	Ji et al. (2022)
*Ne. mikawai*	Cui 11152	KU189773	KU189804	Ji et al. (2022)
*Ne. mikawai*	Dai 12361	KX548975	KX548997	Ji et al. (2022)
***Picipes abieticola***	**Dai 31921**	**PX747571**	**PX747578**	**Present study**
***P. abieticola***	Dai 31927*	**PX747572**	**PX747579**	**Present study**
*P. admirabilis*	Dai 1127	KC572001	—	Unpublished
*P. ailaoshanensis*	Cui 12578	KX900067	KX900182	Ji et al. (2022)
*P. ailaoshanensis*	Cui 12585	KX900068	KX900183	Ji et al. (2022)
*P. americanus*	JV 0509-149	KC572002	KC572041	Ji et al. (2022)
*P. americanus*	JV 0809-104	KC572003	KC572042	Ji et al. (2022)
*P. annularius*	Cui 10123	KX900060	KX900176	Ji et al. (2022)
*P. atratus*	Cui 11289	KX900043	KX900159	Ji et al. (2022)
*P. atratus*	Dai 13375	KX900042	KX900158	Ji et al. (2022)
*P. auriculatus*	Cui 13616	KX900063	KX900179	Ji et al. (2022)
*P. auriculatus*	Yuan 4221	KX900064	KX900180	Ji et al. (2022)
*P. badius*	Cui 10853	KU189780	KU189811	Ji et al. (2022)
*P. badius*	Cui 11136	KU189781	KU189812	Ji et al. (2022)
*P. baishanzuensis*	Cui 11395	KU189763	KU189794	Ji et al. (2022)
*P. baishanzuensis*	Dai 13418	KU189762	KU189793	Ji et al. (2022)
***P. brevisporus***	**Dai 26693**	**PX747573**	**PX747580**	**Present study**
***P. brevisporus***	Dai 26697*	**PX747574**	**PX747581**	**Present study**
*P. brevistipitatus*	Cui 11345	KX900074	KX900188	Ji et al. (2022)
*P. brevistipitatus*	Cui 13652	KX900075	KX900189	Ji et al. (2022)
*P. conifericola*	Cui 9950	KU189783	KU189814	Ji et al. (2022)
*P. conifericola*	Dai 11114	JX473244	KC572061	Ji et al. (2022)
*P. dictyopus*	TENN 59385	AF516561	AJ487945	Ji et al. (2022)
*P. fraxinicola*	Dai 2494	KC572023	KC572062	Ji et al. (2022)
*P. fraxinicola*	Wei 6025	KC572024	KC572063	Ji et al. (2022)
*P. griseus*	Liu 137	PP956601	PP956609	Liu et al. (2024)
*P. hainanensis*	Cui 5327	KU189751	—	Cui et al. (2019)
*P. jiajinensis*	Cui 10748	KU189754	KU189786	Cui et al. (2019)
*P. melanopus*	H 6003449	JQ964422	KC572064	Ji et al. (2022)
*P. melanopus*	MJ 372-93	KC572026	KC572065	Ji et al. (2022)
*P. nigromarginatus*	Cui 8113	KX900062	KX900178	Ji et al. (2022)
*P. pseudovarius*	Cui 10548	NR169904	KU189813	Ji et al. (2022)
*P. pumilus*	Cui 5464	KX851628	KX851682	Ji et al. (2022)
*P. pumilus*	Dai 6705	KX851630	KX851684	Ji et al. (2022)
*P. rhizophilus*	Dai 11599	KC572028	KC572067	Ji et al. (2022)
*P. rhizophilus*	Dai 16082	KX851634	KX851687	Ji et al. (2022)
***P. sinodictyopus***	Dai 20371*	**PX747575**	**PX747582**	**Present study**
***P. sinodictyopus***	**Dai 28935**	**PX747577**	**PX747583**	**Present study**
*P. subdictyopus*	Cui 11220	KX900057	KX900173	Ji et al. (2022)
*P. subdictyopus*	Cui 12539	KX900058	KX900174	Ji et al. (2022)
*P. submelanopus*	Dai 13294	KU189770	KU189801	Ji et al. (2022)
*P. submelanopus*	Dai 13296	KU189771	KU189802	Ji et al. (2022)
*P. subtropicus*	Cui 2662	KU189759	KU189791	Ji et al. (2022)
*P. subtropicus*	Li 1928	KU189758	KU189790	Ji et al. (2022)
*P. subtubaeformis*	Cui 10793	KU189753	KU189785	Ji et al. (2022)
*P. subtubaeformis*	Dai 11870	KU189752	KU189784	Ji et al. (2022)
*P. taibaiensis*	Dai 5741	JX489169	KC572071	Ji et al. (2022)
*P. taibaiensis*	Dai 5746	KX196783	KX196784	Ji et al. (2022)
*P. tibeticus*	Cui 12215	KU189755	KU189787	Ji et al. (2022)
*P. tibeticus*	Cui 12225	KU189756	KU189788	Ji et al. (2022)
*P. tubaeformis*	Niemela 6855	KC572036	KC572073	Ji et al. (2022)
*P. tubaeformis*	JV 0309-1	KC572034	KC572072	Ji et al. (2022)
*P. ulleungus*	Cui 12410	KX900022	KX900142	Ji et al. (2022)
*P. virgatus*	CulTENN11219	AF516581	AJ488122	Ji et al. (2022)
*P. virgatus*	CulTENN11406	AF516582	AJ488122	Ji et al. (2022)
*P. yuxiensis*	CLZhao 971	MZ325828	MZ325830	Bhunjun et al. (2022)
*P. yuxiensis*	CLZhao 980	MZ325827	MZ325829	Bhunjun et al. (2022)
*P. yuxiensis*	CLZhao 6329	MZ325826	—	Bhunjun et al. (2022)
*P. yuxiensis*	CLZhao 6332	MZ325825	—	Bhunjun et al. (2022)
*P. yuxiensis*	CLZhao 6735	MZ325824	—	Bhunjun et al. (2022)
*P. wuyishanensis*	Dai 7409	KX900061	KX900177	Ji et al. (2022)
*Polyporus austrosinensis*	Cui 11140	KX900046	KX900162	Ji et al. (2022)
*Po. austrosinensis*	Cui 11126	KX900045	KX900161	Ji et al. (2022)
*Po. guianensis*	TENN 58404	AF516566	AJ487948	Ji et al. (2022)
*Po. guianensis*	TENN 59093	AF516564	AJ487947	Ji et al. (2022)
*Po. hemicapnodes*	Cui 11259	KX851625	KX851679	Ji et al. (2022)
*Po. hemicapnodes*	Dai 13403	KX851627	KX851681	Ji et al. (2022)
*Po. lamelliporus*	Dai 15106	KX851623	KX851677	Ji et al. (2022)
*Po. lamelliporus*	Dai 12327	KX851622	KX851676	Ji et al. (2022)
*Po. leprieurii*	TENN 58579	AF516567	AJ487949	Ji et al. (2022)
*Po. mangshanensis*	Dai 15151	KX851796	KX851797	Ji et al. (2022)
*Po. parvovarius*	Yuan 6639	KX900049	KX900165	Ji et al. (2022)
*Po. parvovarius*	Dai 13948	KX900050	KX900166	Ji et al. (2022)
*Po. radicatus*	DAOM198916	AF516584	AJ487955	Ji et al. (2022)
*Po. radicatus*	TENN 58831	AF516585	AJ487956	Ji et al. (2022)
*Pseudofavolus cucullatus*	Dai 13584A	KX900071	KX900185	Liu et al. (2024)
*Ps. cucullatus*	WD 2157	AB587637	AB368114	Liu et al. (2024)
*Trametes conchifer*	FP106793sp	JN164924	JN164797	Ji et al. (2022)
*T. elegans*	FP105679sp	JN164944	JN164799	Ji et al. (2022)
*T. polyzona*	Cui 11040	KR605824	KR605767	Ji et al. (2022)

Sequences generated were aligned manually with additional sequences downloaded from GenBank using AliView version 1.27 (Larsson 2014). The final ITS and nLSU datasets were subsequently aligned using MAFFT v.7 under the E-INS-i strategy with no cost for opening gaps and equal cost for transformations (command line: mafft –genafpair –maxiterate 1000, Katoh and Standley (2013) and visualised in AliView. Alignments were spliced and transformed formats in Mesquite v.3.2. (Maddison and Maddison 2017). Multiple sequence alignments were trimmed by trimAI v.1.2 using the -htmlout-gt 0.8 -st option to deal with gaps, when necessary (Capella-Gutierrez et al. 2009).

### Phylogenetic analyses

The two-marker DNA multiple sequence alignment (ITS + nLSU) was used to determine the phylogenetic position of the new species, with *Trametes conchifer* (Schwein.) Pilát, *T. elegans* (Spreng.) Fr. and *T. polyzona* (Pers.) Justo as the outgroup (Ji et al. 2022). The phylogenetic analyses followed the approach of Han et al. (2015) and Zhu et al. (2019). Maximum Likelihood (ML) and Bayesian Inference (BI) analyses were performed, based on the ITS + nLSU datasets.

Sequences were analysed using Maximum Likelihood (ML) with RAxML v.8.2.10 (Stamatakis 2014). Branch supports for all parsimony analyses were estimated by performing 1,000 bootstrap replicates with a heuristic search of 10 random-addition replicates for each bootstrap replicate. Bayesian phylogenetic inference and Bayesian Posterior Probabilities (BPP) were computed with MrBayes 3.2.6 with a GTR + I + G model of DNA substitution and a gamma distribution rate variation across sites (Ronquist and Huelsenbeck 2003). Four Markov chains were run for 1 million generations (two-marker dataset) until the split deviation frequency value was less than 0.01 and trees were sampled every 100 generations. The first 25% of the sampled trees were discarded as burn-in and the remaining ones were used to reconstruct a majority rule consensus and calculate Bayesian Posterior Probabilities (BPP) of the clades. All trees were viewed in FigTree v. 1.4.3 (http://tree.bio.ed.ac.uk/software/figtree/). Branches that received bootstrap support for ML (≥ 75% (ML-BS)) and BPP (≥ 0.95 BPP) were considered as significantly supported. The ML bootstrap (ML) ≥ 50% and BBP (BPP) ≥ 0.90 are presented on topologies from ML analysis, respectively.

## Results

### Molecular phylogeny

The combined two-marker dataset (ITS + nLSU) included sequences from 108 samples representing 65 taxa. The phylogenetic reconstruction performed with Maximum Likelihood (ML) and Bayesian Inference (BI) analyses for the combined dataset showed similar topology and few differences in statistical support. The best model-fit applied in the Bayesian analysis was GTR + I + G, lset nst = 6, rates = invgamma and prset statefreqpr = dirichlet (1, 1, 1, 1). Bayesian analysis and ML analysis resulted in a similar topology to the MP analysis, with an average standard deviation of split frequencies of 0.006865 (BI).

**Figure 1.**
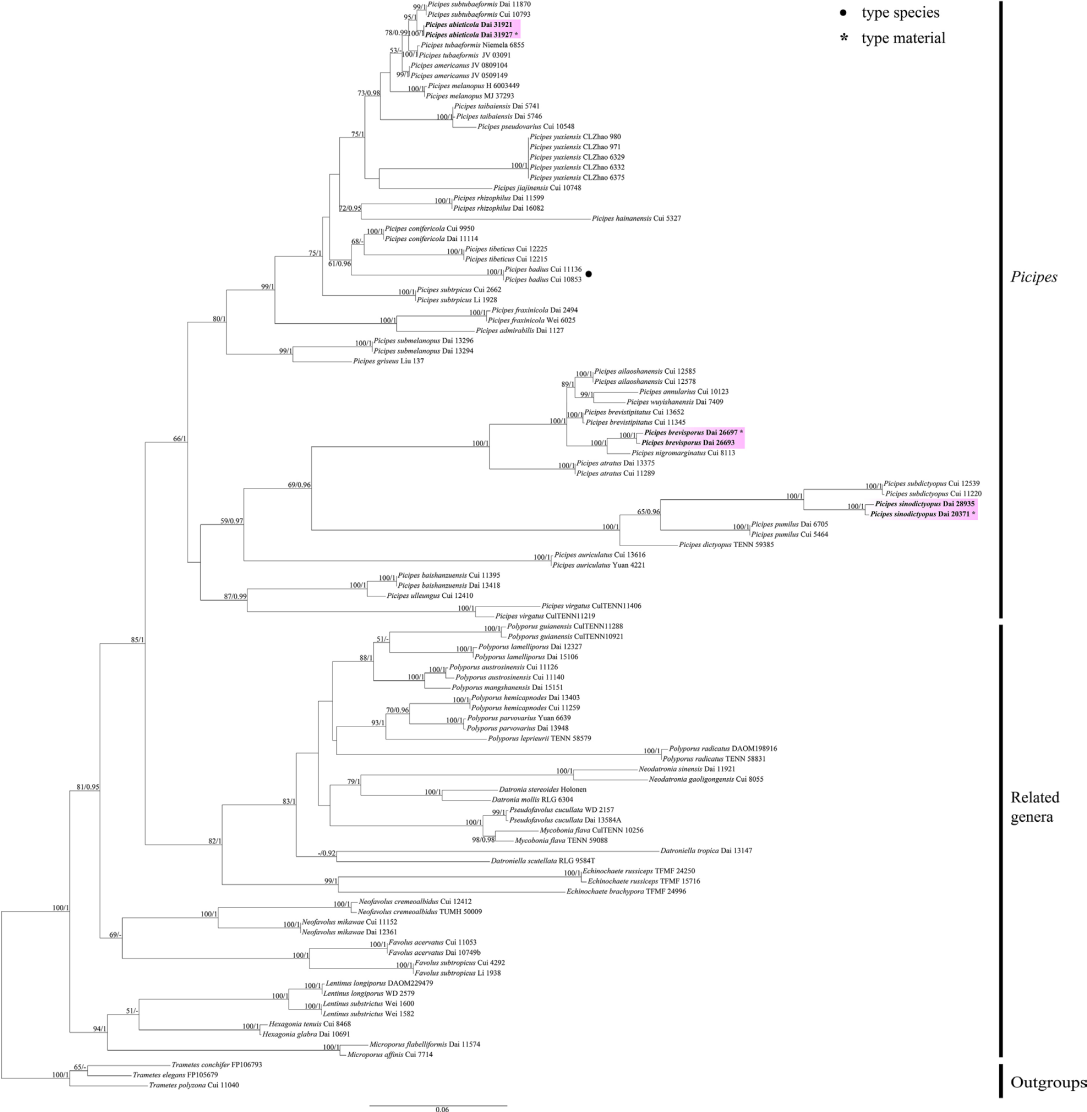
ML analysis of *Picipes*, based on dataset of ITS + nLSU. ML bootstrap values equal to or higher than 50% and Bayesian posterior probabilities values equal to or higher than 0.90 are shown. New taxa are in bold, * represents type material.

The phylogenetic tree inferred from the ITS + nLSU sequences indicated that the three new species belonged to *Picipes* (Fig. 1). In addition, *Picipes abieticola* grouped together with *P. subtubaeformis* J.L. Zhou & B.K. Cui and *P. tubaeformis* with high support (ML = 100, BPP = 1.00); *P. brevisporus* grouped together with *P. nigromarginatus* B.K. Cui, X. Ji & J.L. Zhou with a high support (ML = 100, BPP = 1.00); and *P. sinodictyopus* was sister to *P. subdictyopus* (H. Lee et al.) B.K. Cui, X. Ji & J.L. Zhou with high support (ML = 100, BPP = 1.00).

Based on the ITS and nLSU sequences of the type material, BLAST queries against the authoritative sequences in NCBI yielded alignments, which are shown in Table 2.

**Table 2.** Based on the ITS and nLSU sequences of the type material, BLAST queries against the authoritative sequences in NCBI yielded alignments.

Species Name	Marker	Top Match Species	GenBank Accession No.	Max Score	Total Score	Query Cover	E-value	Identity
*Picipes abieticola*	ITS	*P. tubaeformis*	KC572034	1002	1002	90%	0.0	97.77%
	nLSU	*P. subtubaeformis*	KU189784	2459	2459	100%	0.0	99.63%
*P. brevisporus*	ITS	*P. nigromarginatus*	NR184461	889	889	88%	0.0	92.00%
	nLSU	*P. brevistipitatus*	NG148947	1411	1411	99%	0.0	98.74%
*P. sinodictyopus*	ITS	*P. subdictyopus*	NR158925	767	767	77%	0.0	92.74%
	nLSU	*P. subdictyopus*	PP956609	2357	2357	99%	0.0	97.67%

### Taxonomy

#### Picipes abieticola

Taxon classificationFungiPolyporalesPolyporaceae

K.Y. Luo, Y.C. Dai & Y.J. Cui sp. nov.

3876E9CC-E238-5714-A364-2239B5705B79

861751

Figs 2, 3

##### Holotype.

China • Xizang Autonomous Region, Yadong County, Pasha Falls to Yadong Customs. GPS coordinates: 27°25'N, 88°55'E; altitude: 2800 m a.s.l. On fallen branch of *Abies fabri*, 18 October 2024, *Dai 31927* (BJFC052186).

**Figure 2.**
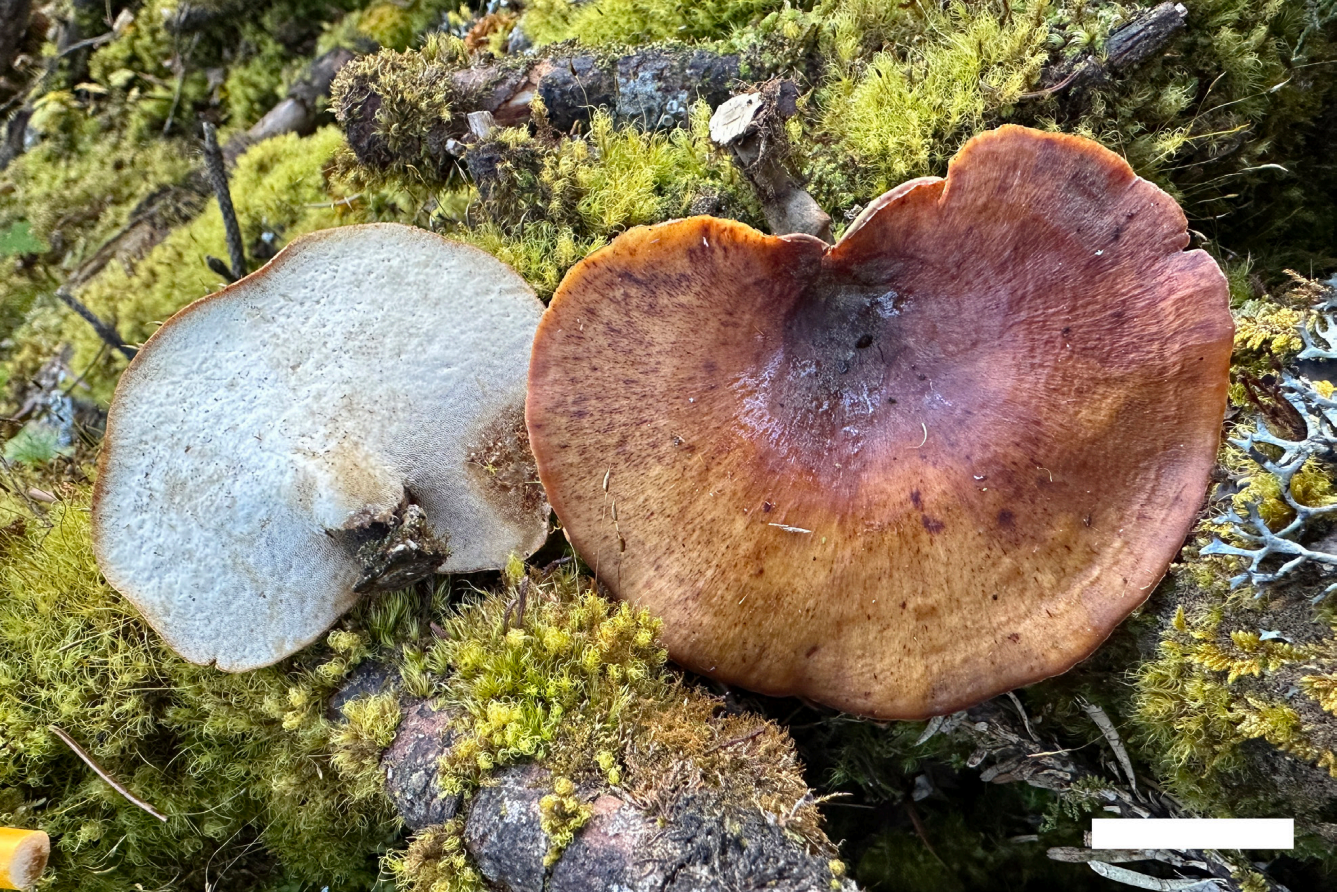
Basidiomata of *Picipes abieticola* (Holotype, Dai 31927). Scale bar: 2 cm.

##### Diagnosis.

*Picipes abieticola* is identified by eccentrically stipitate, fan-shaped pilei, circular to subcircular pores (5–7/mm); a dimitic hyphal structure and cylindrical basidiospores with dimensions of 7.5–9.3 × 2.8–3.5 µm.

**Figure 3.**
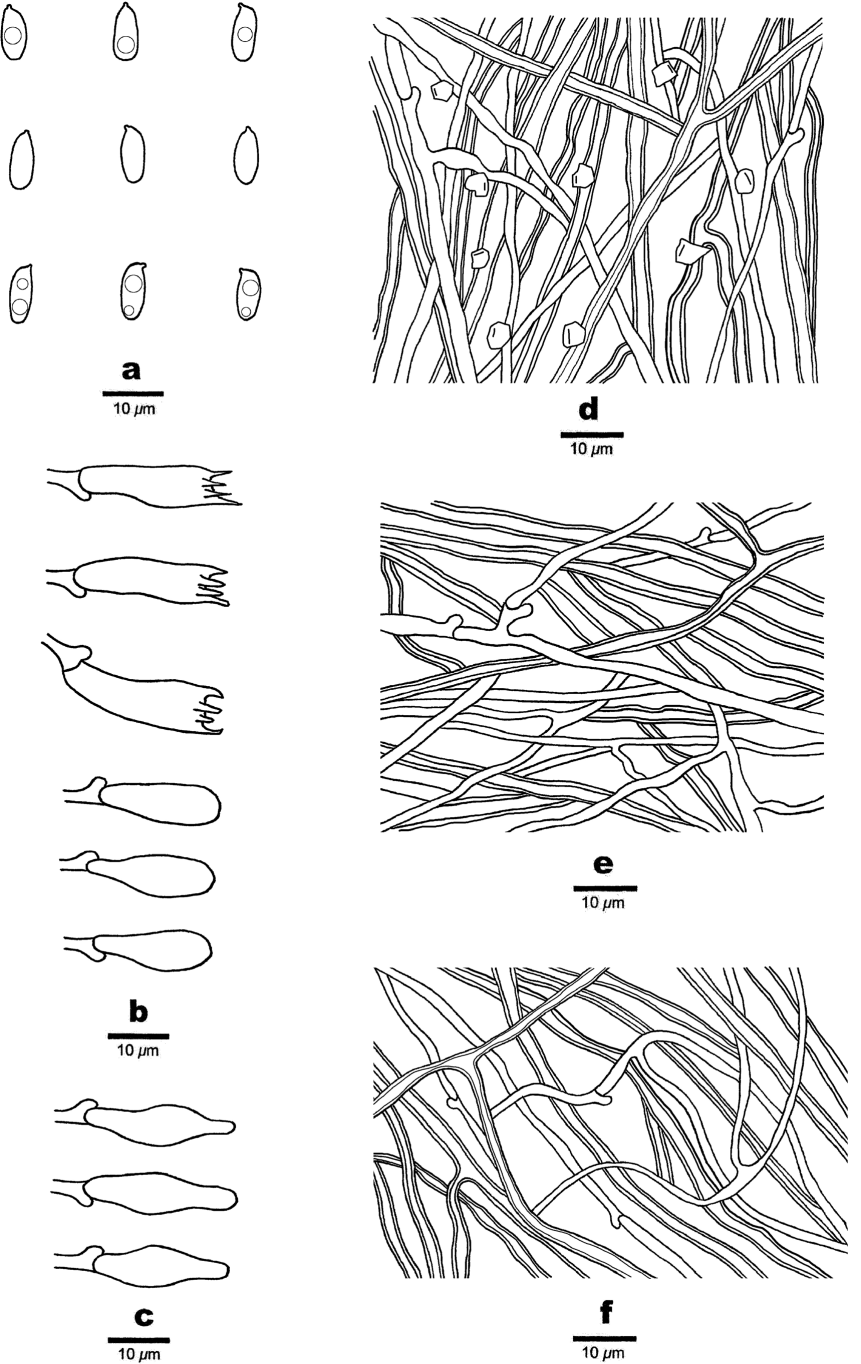
Microscopic structures of *Picipes abieticola* (Holotype, Dai 31927). **a**. Basidiospores; **b**. Basidia and basidioles; **c**. Cystidioles; **d**. Hyphae from trama; **e**. Hyphae from context; **f**. Hyphae from stipe.

##### Etymology.

*Abieticola* (Lat.) refers to the species inhabiting *Abies*.

##### Description.

Basidiomata annual, eccentrically stipitate, solitary, coriaceous and without odour or taste when fresh, hard corky when dry. Pilei fan-shaped, projecting up to 7 cm long, 5 cm wide, and 2 mm thick at base. Pileal surface blackish-red to black, darkening to more or less the centre when fresh, becoming brownish-red to yellowish-brown towards the margin when dry, glabrous, with radially aligned stripes; margin incurved upon drying. Pore surface white when fresh, light ivory to buff when dry, shining; pores circular to subcircular, 5–7 per mm; dissepiments thin, entire. Context light ivory to buff when dry, hard corky upon drying, up to 0.5 mm thick. Tubes concolorous with context, up to 1.5 mm long. Stipe very short, bearing a black cuticle, up to 1.5 cm long and 8 mm in diam.

Hyphal structure: Hyphal system dimitic; generative hyphae bearing clamp connections; skeletal-binding hyphae IKI–, slightly CB+; tissues unchanged in KOH.

Context: Generative hyphae infrequent, colourless, thin-walled, moderately branched, 2–3 μm in diam.; skeletal-binding hyphae dominant, colourless, thick-walled with a wide to narrow lumen, occasionally branched, interwoven, 3–4 μm in diam.

Tubes: Generative hyphae infrequent, colourless, thin-walled, occasionally branched, 1.8–2.7 μm in diam.; skeletal-binding hyphae colourless, thick-walled with a wide to narrow lumen, occasionally branched, interwoven, 3–4 μm in diam. Irregular crystals present. Cystidia absent, cystidioles subulate, 20–25 × 4–6 μm. Basidia mostly clavate, with a basal clamp connection and four sterigmata, 17–25 × 5–8 μm; basidioles in shape similar to basidia, but slightly smaller.

Stipe: Generative hyphae infrequent, colourless, thin-walled, moderately branched, 1.5–2 μm in diam.; skeletal-binding hyphae dominant, colourless, thick-walled with a wide to narrow lumen, moderately branched, interwoven, 3.5–5.5 μm in diam.

Basidiospores—Cylindrical, hyaline, thin-walled, smooth, some with one or two guttules, IKI–, CB–, (7–)7.5–9.3(–9.5) × (2.7–)2.8–3.5(–3.8) µm, L = 8.51 µm, W = 3.18 µm, Q = 2.64–2.72 (n = 60/2).

##### Type of rot.

White rot.

##### Additional specimen (paratype) examined.

China • Xizang Autonomous Region, Yadong County, Pasha Falls to Yadong Customs. GPS coordinates: 27°25'N, 88°55'E; altitude: 2800 m a.s.l. On fallen branch of *Abies fabri*, 18 October 2024, *Dai 31921* (BJFC052180).

#### Picipes brevisporus

Taxon classificationFungiPolyporalesPolyporaceae

K.Y. Luo, Y.C. Dai, Y.J. Cui & Xin Zhang sp. nov.

6BCC9C04-6205-5D0F-B266-330500A0227B

861752

Figs 4, 5

##### Holotype.

China • Xizang Autonomous Region, Motuo County, the road 219 from Motuo County to Jiefang Bridge. GPS coordinates: 29°18'N, 95°18'E; altitude: 1000 m a.s.l. On dead *Miscanthus*, 24 October 2023, *Dai 26697* (BJFC 044247).

**Figure 4.**
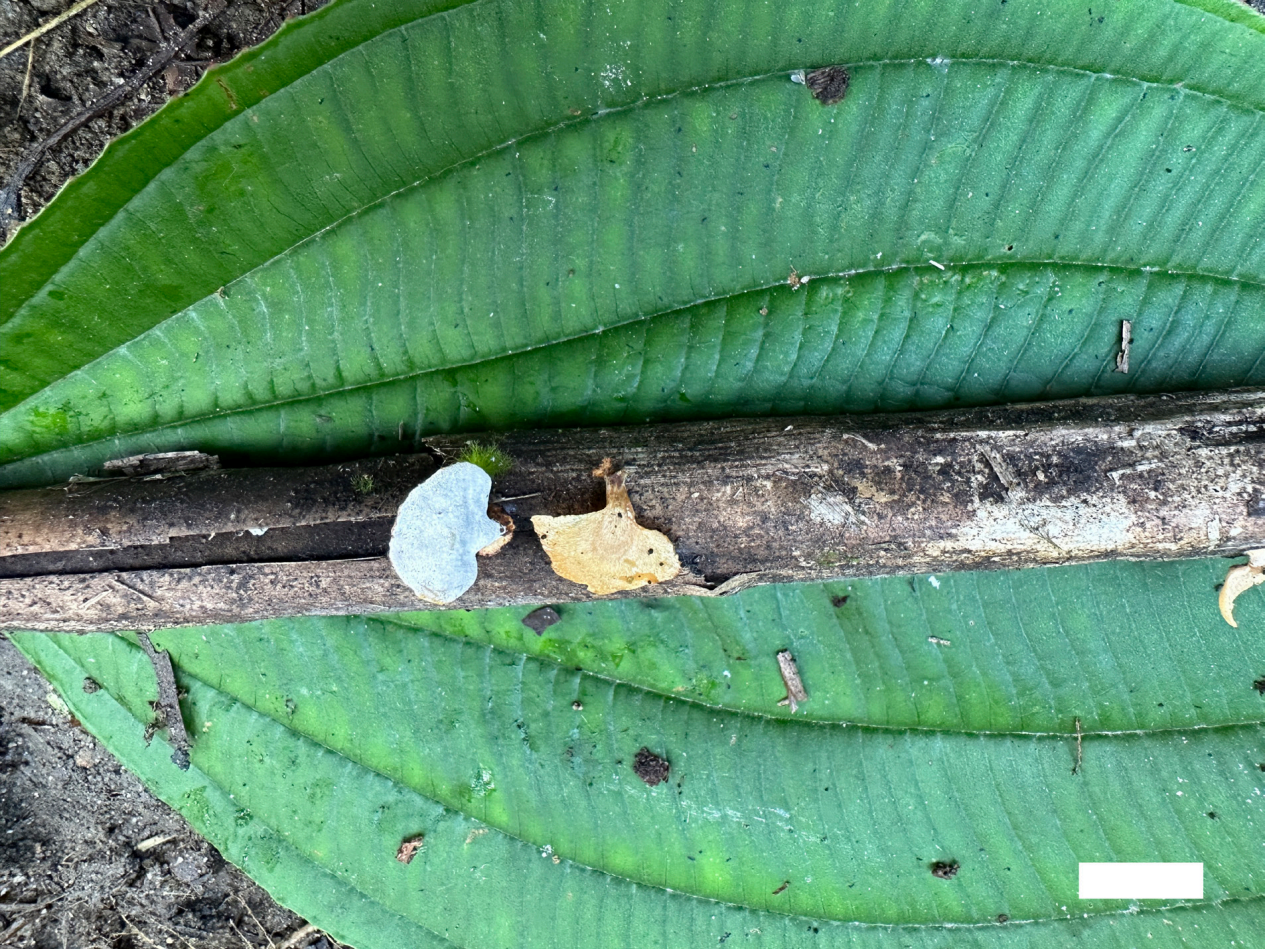
Basidiomata of *Picipes brevisporus* (Holotype, Dai 26697). Scale bar: 1 cm.

##### Diagnosis.

*Picipes brevisporus* is identified by laterally stipitate, glabrous, with radially aligned stripes pileal surface, short stipe; bearing clamp connections on generative hyphae, subulate cystidioles and oblong ellipsoid basidiospores with dimensions of 3.8–5.7 × 2.3–3.1 µm.

**Figure 5.**
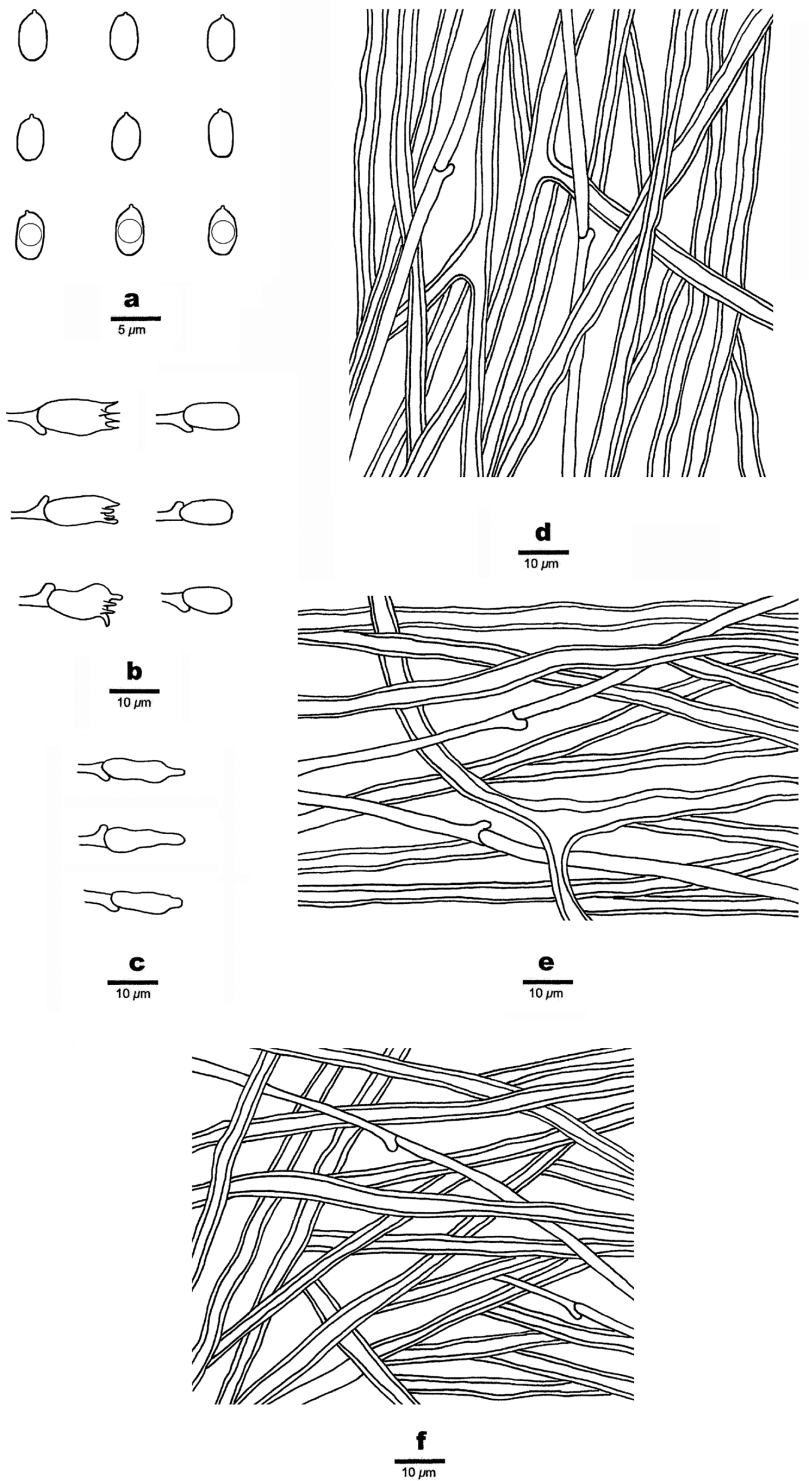
Microscopic structures of *Picipes brevisporus* (Holotype, Dai 26697). **a**. Basidiospores; **b**. Basidia and basidioles; **c**. Cystidioles; **d**. Hyphae from trama; **e**. Hyphae from context; **f**. Hyphae from stipe.

##### Etymology.

*Brevisporus* (Lat.) refers to the species having short basidiospores in the genus *Picipes*.

##### Description.

Basidiomata annual, laterally stipitate, solitary, coriaceous and without odour or taste when fresh, hard corky when dry. Pilei auricular to fan-shaped, about 1–1.5 cm long, 0.4–0.6 cm wide and up to 1 mm thick at base. Pileal surface yellow-brownish to brownish when fresh, becoming brown to black towards the margin when dry, glabrous, with radially aligned stripes; margin straight. Pore surface bluish-grey when fresh, light ivory to buff when dry; pores circular to subcircular, 7–9 per mm; dissepiments thin, entire. Context light ivory to buff when dry, hard corky upon drying, up to 0.5 mm thick. Tubes concolorous with pore surface, up to 0.5 mm long. Stipe short, bearing a black cuticle, up to 2 mm long and 2 mm in diam.

Hyphal structure: Hyphal system dimitic; generative hyphae bearing clamp connections; skeletal hyphae IKI–, slightly CB+; tissues unchanged in KOH.

Context: Generative hyphae infrequent, colourless, thin-walled, rarely branched, 2–3 μm in diam.; skeletal-binding hyphae dominant, colourless, thick-walled, occasionally branched, interwoven, 3.5–4.5 μm in diam.

Tubes: Generative hyphae dominant, colourless, thin-walled, rarely branched, 2–3 μm in diam.; skeletal-binding hyphae colourless, thick-walled, moderately branched, interwoven, 3–4.5 μm in diam. Cystidia absent, cystidioles subulate, 12–16 × 3–4 μm. Basidia more or less barrel-shaped, with a basal clamp connection and four sterigmata, 10–15 × 5–7 μm; basidioles in shape similar to basidia, but slightly smaller.

Stipe: Generative hyphae infrequent, colourless, thin-walled, rarely branched, 2–3 μm in diam.; skeletal-binding hyphae dominant, colourless, thick-walled, rarely branched, interwoven, 3.5–4.5 μm in diam.

Basidiospores: Short oblong ellipsoid, hyaline, thin-walled, smooth, some with one large guttule, IKI–, CB–, (3.5–)3.8–5.7(–6.6) × (2.0–)2.3–3.1(–3.4) µm, L = 4.75 µm, W = 2.67 µm, Q = 1.71–1.85 (n = 60/2).

##### Type of rot.

White rot.

##### Additional specimen (paratype) examined.

China • Xizang Autonomous Region, Motuo County, the Road 219 from Motuo County to Jiefang Bridge. GPS coordinates: 29°18'N, 95°18'E; altitude: 1000 m a.s.l. On dead *Miscanthus*, 24 October 2023, *Dai 26693* (BJFC 044243).

#### Picipes sinodictyopus

Taxon classificationFungiPolyporalesPolyporaceae

K.Y. Luo, Y.C. Dai & G.Y. Zeng sp. nov.

A0FFC84A-0FBD-528D-A4AC-A50ED60A91FE

861753

Figs 6, 7

##### Holotype.

China • Yunnan Province, Wuding County, Shizishan Nature Reserve. GPS coordinates: 25°33'N, 102°21'E; altitude: 2000 m a.s.l. On dead branch of *Litocarpus*, 15 August 2019, *Dai 20371* (BJFC 032039).

**Figure 6.**
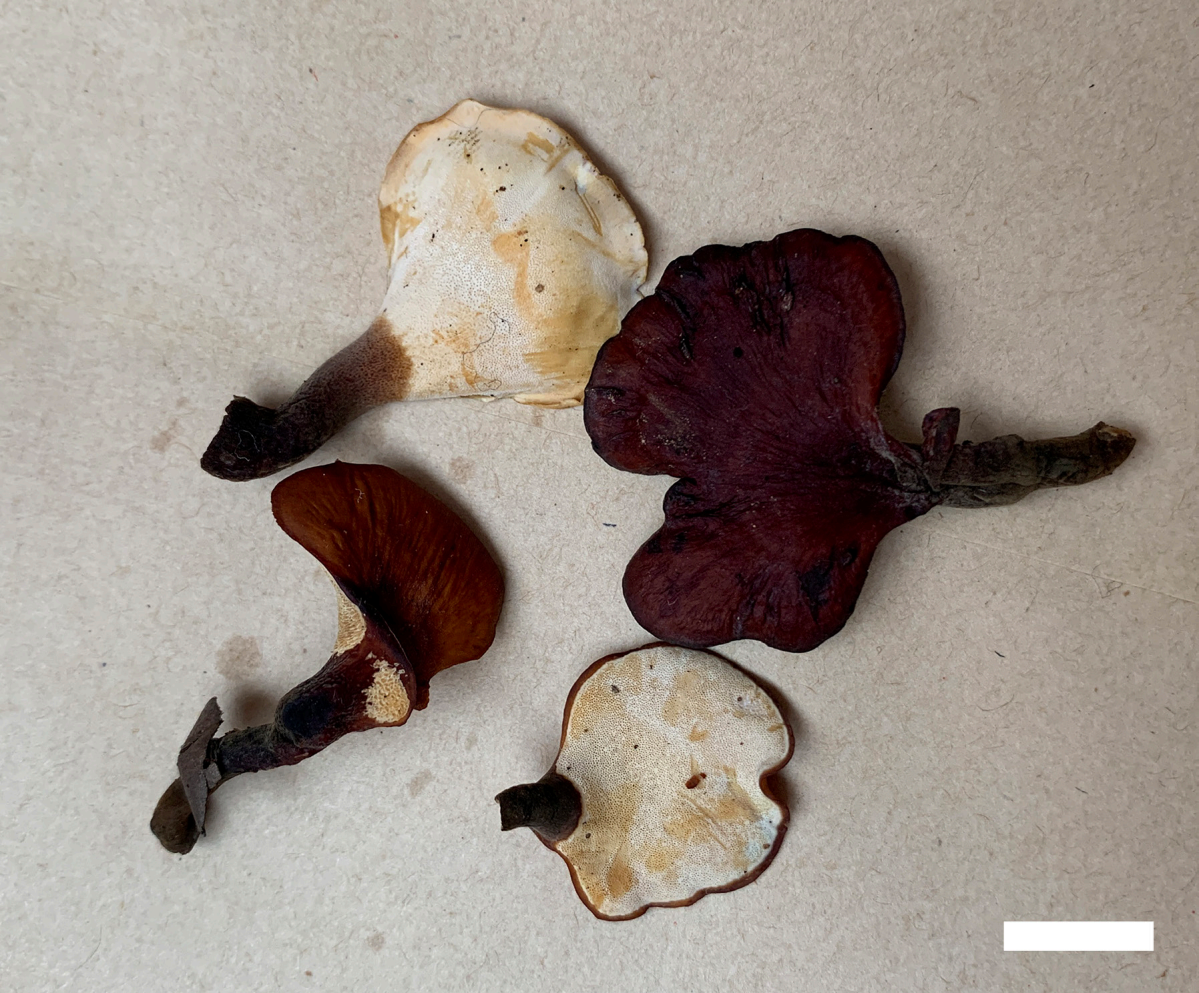
Basidiomata of *Picipes sinodictyopus* (Holotype, Dai 20371). Scale bar: 1 cm.

##### Diagnosis.

*Picipes sinodictyopus* is identified by coriaceous and without odour or taste basidiomata, cream to buff when fresh, olivaceous-buff when dry pore surface, angular pores, long stipe; infrequent generative hyphae in context, tubes and stipe and cylindrical, thin-walled basidiospores with dimensions of 5.8–7.3 × 2.3–2.8 µm.

**Figure 7.**
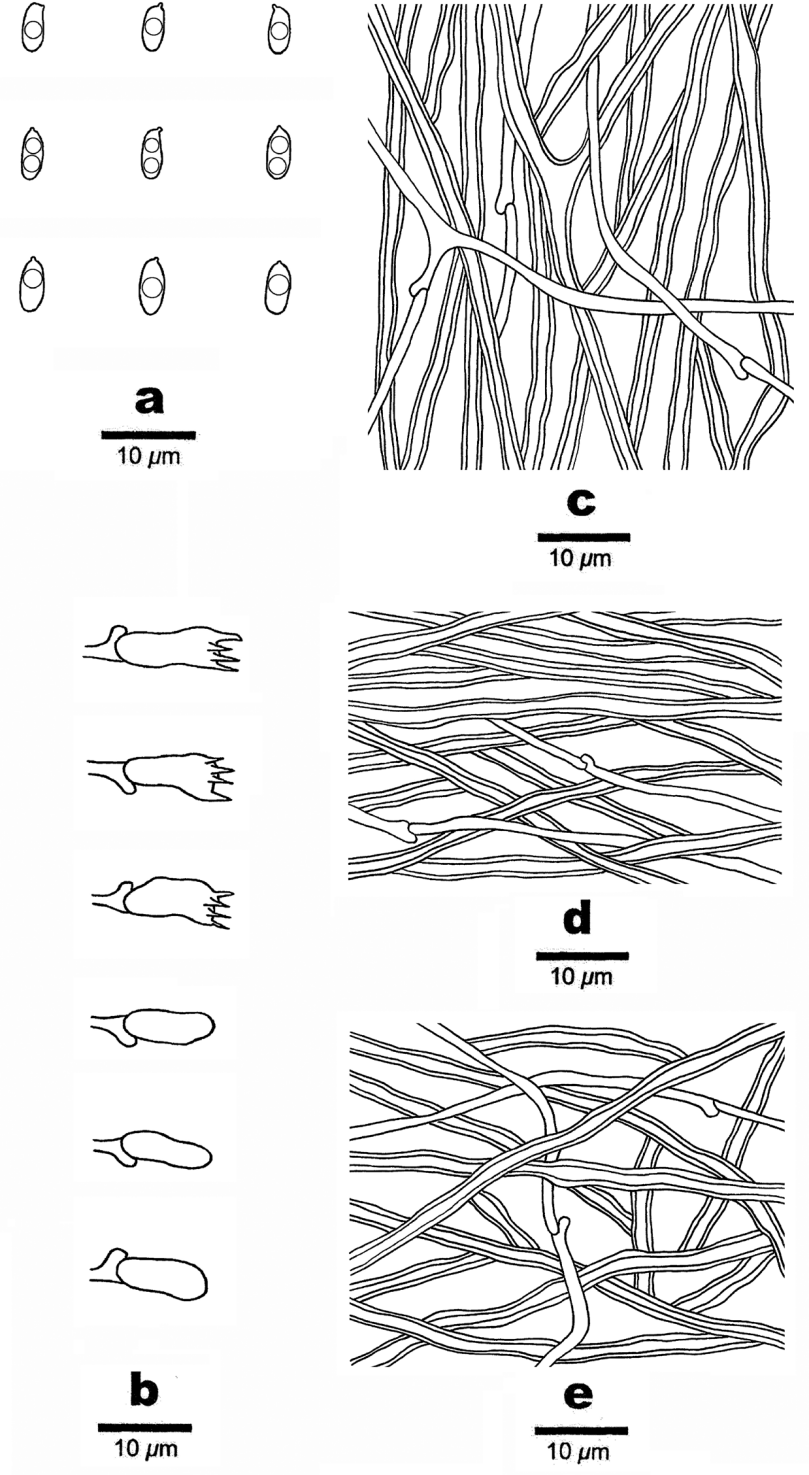
Microscopic structures of *Picipes sinodictyopus* (Holotype, Dai 20371). **a**. Basidiospores; **b**. Basidia and basidioles; **c**. Hyphae from trama; **d**. Hyphae from context; **e**. Hyphae from stipe.

##### Etymology.

*Sinodictyopus* (Lat.) refers to the species similar to *Picipes dictyopus*, but having a distribution in China.

##### Description.

Basidiomata annual, laterally stipitate, solitary, coriaceous and without odour or taste when fresh, hard corky when dry. Pilei auricular to fan-shaped, about 2–2.5 cm long, 1.5–2 cm wide and up to 2 mm thick at base. Pileal surface reddish-brown to dark brown when fresh, becoming brown to reddish-brown when dry, glabrous; margin straight. Pore surface cream to buff when fresh, olivaceous-buff when dry; pores angular, 4–6 per mm; dissepiments thin, entire. Context light ivory to cream when dry, woody hard upon drying, up to 1 mm thick. Tubes concolorous with pore surface, up to 1 mm long. Stipe long, bearing a black cuticle, up to 1.5 cm long and 0.6 cm in diam.

Hyphal structure: Hyphal system dimitic; generative hyphae bearing clamp connections; skeletal-binding hyphae IKI–, CB+; tissues unchanged in KOH.

Context: Generative hyphae infrequent, colourless, thin-walled, rarely branched, 1–2 μm in diam.; skeletal-binding hyphae dominant, colourless, thick-walled, rarely branched, interwoven, 2.5–4 μm in diam.

Tubes: Generative hyphae infrequent, colourless, thin-walled, occasionally branched, 1.5–3 μm in diam; skeletal-binding hyphae colourless, thick-walled, occasionally branched, interwoven, 3–4.5 μm in diam. Cystidia and cystidioles absent. Basidia subclavate, with a basal clamp connection and four sterigmata, 12–22 × 4–8 μm; basidioles in shape similar to basidia, but slightly smaller.

Stipe: Generative hyphae infrequent, colourless, thin-walled, rarely branched, 1–2 μm in diam.; skeletal-binding hyphae dominant, colourless, thick-walled, rarely branched, interwoven, 3–4 μm in diam.

Basidiospores: Cylindrical, hyaline, thin-walled, smooth, some with one or two medium guttules, IKI–, CB–, (5.2–)5.8–7.3(–7.4) × (2.1–)2.3–2.8(–3.2) µm, L = 6.47 µm, W = 2.52 µm, Q = 2.57 (n = 30/1) (the specimen Dai 28935 is sterile).

##### Type of rot.

White rot.

##### Additional specimen (paratype) examined.

China • Guangxi Autonomous Region, Jinxiu County, Liugang, Daling Village. GPS coordinates: 23°59'N, 110°05'E; altitude: 800 m a.s.l. On rotten angiosperm wood, 15 July 2024, *Dai 28935* (BJFC049194).

## Discussion

Phylogenetically, three new species are nested in *Picipes* clade, based on ITS + nLSU topology (Fig. 1). Amongst them, *P. abieticola* grouped together with *P. subtubaeformis* and *P. tubaeformis*; however, *P. subtubaeformis* is delimited from *P. abieticola* by having shorter basidiospores (5.7–6.8 × 2.7–3.1 μm vs. 7.5–9.3 × 2.8–3.5 µm, Zhou et al. (2016); *P. tubaeformis* differs from *P. abieticola* by having circular to infundibuliform pilei and shorter basidiospores (6–7.8 × 2.3–3.2 μm vs. 7.5–9.3 × 2.8–3.5 µm, Dai et al. (1996). *Picipes brevisporus* grouped together with *P. nigromarginatus*; however, *P. nigromarginatus* is different from *P. brevisporus* by having larger pilei (2.2 × 3.4 cm vs. 1.5 × 0.6 cm), larger stipe (5 × 4 mm vs. 2 × 2 mm) and longer basidiospores (5.6–6.9 × 2.3–3 μm vs. 3.8–5.7 × 2.3–3.1 µm, Ji et al. (2022). *Picipes sinodictyopus* was sister to *P. subdictyopus*, but *P. subdictyopus* differs from *P. sinodictyopus* by its smaller pores (8–10 per mm vs. 4–6 per mm Tibpromma et al. (2017).

Morphologically, *Picipes abieticola* resembles *P. dictyopus* (Mont.) B.K. Cui, X. Ji & J.L. Zhou and *P. subtropicus* J.L. Zhou & B.K. Cui by sharing similar pores (5–7 per mm). However, *P. dictyopus* is different from *P. abieticola* by its longer tubes (3 mm vs. 1.5 mm) and longer stipe (5 cm vs. 1.5 cm) and growing on angiosperm wood in tropical and subtropical areas (Ryvarden and Johansen 1980); *P. subtropicus* is distinguished from *P. abieticola* by its smaller stipe (5 × 5 mm vs. 15 × 8 mm) and smaller basidiospores (5.1–6.2 × 2.2–2.7 μm vs. 7.5–9.3 × 2.8–3.5 µm, Zhou et al. (2016).

*Picipes brevisporus* resembles *P. badius*, *P. baishanzuensis* J.L. Zhou & B.K. Cui and *P. conifericola* (H.J. Xue & L.W. Zhou) J.L. Zhou & B.K. Cui by sharing subulate cystidioles. However, *P. badius* is different from *P. brevisporus* by larger basidiospores (5.5–8 × 3–3.8 μm vs. 3.8–5.7 × 2.3–3.1 µm) and lacking clamp connections (Cui et al. 2019); *P. baishanzuensis* is distinguished from *P. brevisporus* by having larger pores (3–6 per mm vs. 7–9 per mm) and longer basidiospores (6.6–7.9 × 2.5–3.1 μm vs. 3.8–5.7 × 2.3–3.1 µm) and growing on angiosperm wood (Cui et al. 2019); *P. conifericola* is different from *P. brevisporus* by its larger basidiomata (7 cm diam. vs. 1–1.5 cm long, 0.4–0.6 cm wide) and longer basidiospore, and growing on conifers (6.5–8.3 × 2.9–3.5 μm vs. 3.8–5.7 × 2.3–3.1 µm, Cui et al. (2019).

*Picipes sinodictyopus* is similar to *P. annularius* B.K. Cui et al., *P. pseudovarius* J.L. Zhou & B.K. Cui and *P. taibaiensis* (Y.C. Dai) J.L. Zhou & B.K. Cui by lacking cystidia and cystidioles. However, *P. annularius* is distinguished from *P. sinodictyopus* by having buff pileal surface and smaller stipe (4 mm × 3.5 mm vs. 15 mm × 6 mm, Ji et al. (2022); *P. pseudovarius* is different from *P. sinodictyopus* by its shorter stipe (5 mm vs. 15 mm) and longer basidiospores (7.7–9.3 × 2.6–3.4 μm vs. 5.8–7.3 × 2.3–2.8 µm, Cui et al. (2019); *P. taibaiensis* is distinguished from *P. sinodictyopus* by having shorter stipe (5 × 4 mm vs. 15 × 6 mm) and larger basidiospores (7.6–10.8 × 3.3–4.1 μm vs. 5.8–7.3 × 2.3–2.8 µm, Cui et al. (2019).

This paper enriches our knowledge of fungal diversity in the genus *Picipes* and it is likely that more new taxa will be found with further fieldwork and molecular analyses. There is still a lot of room to explore the diversity of *Picipes* species in China.

### Key to species of *Picipes* in China

**Table:** 

1	Growing on grass roots	***P. rhizophilus***
–	Growing on woods or ground	**2**
2	Generative hyphae bearing simple septa	**3**
–	Generative hyphae bearing clamp connections	**4**
3	Pores 2–3 per mm; basidiospores 8–10 × 3–3.9 µm	***P. submelanopus***
–	Pores 5–6 per mm; basidiospores 6.5–8 × 3–3.8 µm	***P. badius***
4	Growing on coniferous woods	**5**
–	Growing on hardwoods or monocotyledon	**9**
5	Pores 2–5 per mm; cystidioles absent	***P. pseudovarius***
–	Pores ≥ 6 per mm; cystidioles present	**6**
6	Basidiospores cylindrical, usually > 6 µm in length	**7**
–	Basidiospores oblong to cylindrical, usually < 6 µm in length	**8**
7	Pores 7–10 per mm; stipe slender, up to 5 cm	***P. conifericola***
–	Pores 5–7 per mm; stipe short, up to 1.5 cm	***P. abieticola***
8	Basidiospores mainly oblong; growing in plateau temperate regions	***P. tibeticus***
–	Basidiospores mainly cylindrical; growing in subtropical regions	***P. jiajinensis***
9	Pileal surface grey	***P. griseus***
–	Pileal surface other colour rather than grey	**10**
10	Pores ≥ 7 per mm	**11**
–	Pores < 7 per mm	**15**
11	Cystidioles absent	***P. pumilus***
–	Cystidioles subulate	**12**
12	Pileal surface azonate	***P. subtropicus***
–	Pileal surface zonate	**13**
13	Growing on monocotyledon	***P. brevisporus***
–	Growing on angiosperm	**14**
14	Stipe up to 1.5 cm long and 1 cm in diam.	***P. atratus***
–	Stipe up to 5 mm long and 4 mm in diam.	***P. nigromarginatus***
15	Cystidioles absent	**16**
–	Cystidioles present	**22**
16	Stipe central	**17**
–	Stipe lateral	**18**
17	Basidiospores with one to two guttules	***P. subdictyopus***
–	Basidiospores without guttules	***P. ulleungus***
18	Stipe without a black cuticle	**19**
–	Stipe with a black cuticle	**20**
19	Stipe up to 4 mm long and 3.5 mm in diam.	***P. annularius***
–	Stipe up to 2 cm long and 1.5 cm in diam.	***P. fraxinicola***
20	Basidia ≥ 25 µm in length	***P. yuxiensis***
–	Basidia < 25 µm in length	**21**
21	Pores circular to subcircular	***P. auriculatus***
–	Pores angular	***P. sinodictyopus***
22	Stipe up to 8 cm	***P. admirabilis***
–	Stipe up to 2.5 cm	**23**
23	Cystidioles absent	***P. taibaiensis***
–	Cystidioles present	**24**
24	Stipe without a black or terra-brown cuticle	***P. brevistipitatus***
–	Stipe with a black or terra-brown cuticle	**25**
25	Stipe slender	***P. baishanzuensis***
–	Stipe short or with a flattened base	**26**
26	Pore surface grey-beige or pearl-beige upon drying	**27**
–	Pore surface tan or buff upon drying	**28**
27	Hyphae in cuticle bearing clamp connections	***P. ailaoshanensis***
–	Hyphae in cuticle with simple septa	***P. wuyishanensis***
28	Basidia < 16 µm in length	***P. hainanensis***
–	Basidia > 16 µm in length	***P. subtubaeformis***

## Supplementary Material

XML Treatment for Picipes abieticola

XML Treatment for Picipes brevisporus

XML Treatment for Picipes sinodictyopus

## Additional information

### Use of AI

No use of AI was reported.

### Author ORCIDs

Kai-Yue Luo https://orcid.org/0000-0001-6145-4864

Yu-Jin Cui https://orcid.org/0009-0000-8706-6771

Si-Yi Feng https://orcid.org/0009-0008-7741-6879

Xin Zhang https://orcid.org/0009-0005-8363-7852

Yu-Cheng Dai https://orcid.org/0000-0002-6523-0320

Guang-Yu Zeng https://orcid.org/0009-0009-8609-1612

### Data availability

All of the data that support the findings of this study are available in the main text.
